# Allele-specific differences in ryanodine receptor 1 mRNA expression levels may contribute to phenotypic variability in malignant hyperthermia

**DOI:** 10.1186/1750-1172-5-10

**Published:** 2010-05-19

**Authors:** Hilbert Grievink, Kathryn M Stowell

**Affiliations:** 1 Institute of Molecular BioSciences, Massey University, Private Bag 11-222, Palmerston North, New Zealand

## Abstract

**Background:**

Malignant hyperthermia (MH) is a dominantly inherited skeletal muscle disorder that can cause a fatal hypermetabolic reaction to general anaesthetics. The primary locus of MH (MHS1 locus) in humans is linked to chromosome 19q13.1, the position of the gene encoding the ryanodine receptor skeletal muscle calcium release channel (RyR1).

**Methods:**

In this study, an inexpensive allele-specific PCR (AS-PCR) assay was designed that allowed the relative quantification of the two RyR1 transcripts in heterozygous samples found to be susceptible to MH (MHS). Allele-specific differences in RyR1 expression levels can provide insight into the observed variable penetrance and variations in MH phenotypes between individuals. The presence/absence of the H4833Y mutation in *RYR*1 transcripts was employed as a marker that allowed discrimination between the two alleles.

**Results:**

In four skeletal muscle samples and two lymphoblastoid cell lines (LCLs) from different MHS patients, the wild type allele was found to be expressed at higher levels than the mutant RyR1 allele. For both LCLs, the ratios between the wild type and mutant *RYR*1 alleles did not change after different incubation times with actinomycin D. This suggests that there are no allele-specific differences in RyR1 mRNA stability, at least in these cells.

**Conclusion:**

The data presented here revealed for the first time allele-specific differences in *RYR*1 mRNA expression levels in heterozygous MHS samples, and can at least in part contribute to the observed variable penetrance and variations in MH clinical phenotypes.

## Background

Malignant hyperthermia (MH) [MIM no. 145600], first described by Denborough and Lovell [1], is a dominantly inherited skeletal muscle disorder that predisposes susceptible individuals to a potentially fatal reaction during general anaesthesia [2]. Besides this toxic response to anaesthetics, in rare circumstances MH may also be triggered in susceptible individuals by severe exercise in hot conditions, infections, neuroleptic drugs, or overheating in infants [3]. The incidence of MH has been estimated to be as high as 1:2000 [4]. The true genetic predisposition of the condition is difficult to ascertain, since many fulminant MH reactions occur for the first time in patients who have previously undergone uneventful anaesthesia [5]. The clinical signs of an MH reaction are highly variable and are caused by a hypermetabolic state with muscle rigidity, metabolic acidosis, rhabdomyolysis, tachycardia, and/or an increase in body temperature [6]. The primary locus of MH (MHS1 locus) in humans is linked to chromosome 19q13.1, the position of the gene encoding the ryanodine receptor skeletal muscle calcium release channel (RyR1) [7,8]. Besides MH, the *RYR1 *gene [MIM no. 180901, GenBank: NM_000540] is also linked to congenital myopathies namely, central core disease (CCD) [MIM no. 117000] and multi minicore disease (MmD) [MIM no. 255320].

Due to the lack of clinical symptoms under normal conditions and the heterogeneity of MH, the accepted 'gold standard' diagnosis for susceptibility to MH (MHS) is by *in vitro *contracture test (IVCT) [9]. Additionally, various functional assays [10-13] and genetic tests [14] have been developed to aid in the understanding and diagnosis of MH. Several studies reported discordance between MH phenotypes and genotypes [15-18]. Different causative MH mutations have been found to differentially affect muscle contraction in IVCT and Ca^2+ ^release in functional assays, respectively [19-21]. Girard *et al*. [20] showed that halothane-induced changes in intracellular calcium concentrations of skeletal muscle cells, is not simply mutation specific. It was also found to be individual specific. These findings indicate that besides the specific mutation, a variety of other genetic and environmental factors, such as muscle quality, mutation penetrance, and variations in gene expression might also play roles in the observed variations in MH phenotypes.

Polymorphisms and variations in gene expression provide the genetic basis for variation in populations. Several recent studies revealed that allelic variations in gene expression are common in the human genome even among non-imprinted autosomal genes and can follow a mendelian inheritance [22-24]. This suggests that other mechanisms besides epigenetic phenomena can lead to allelic variations and consequently may contribute to the observed variations in MH phenotypes between individuals.

This study aimed to determine if there are allele-specific differences in *RYR*1 messenger RNA (mRNA) expression levels, in heterozygous MHS samples. The causative H4833Y MH mutation was used as a marker to allow discrimination between the two *RYR*1 transcripts [11]. Measuring the expression of each of the two alleles simultaneously in the one target tissue is optimal for detecting *cis*-acting differences as each allele serves as an internal control for the other. Any *trans*-acting effects of environmental conditions that differentially influence gene expression among samples should not interfere. Plasmid constructs, representing the wild type and mutant 4833 *RYR*1 alleles, were used for assay validation. Four MHS skeletal muscle tissues were screened to determine if there were allele-specific differences in mRNA expression levels between the wild type and mutant RyR1 alleles. Two lymphoblastoid cell lines (LCLs) derived from blood of MHS individuals were used in mRNA stability assays to determine possible allelic-specific differences in *RYR*1 mRNA stabilities.

## Methods

### Patient samples

Four frozen MHS skeletal muscle tissues stored subsequent to biopsy for IVCT analysis and two Epstein-Barr virus (EBV)-immortalized LCLs derived from blood of MHS individuals carrying the causative H4833Y MH mutation, were used after informed consent from participating subjects. Note that both skeletal muscle biopsies and LCLs were not available from any single individual. Thus, all four skeletal muscle biopsies and two LCLs were obtained from six different MHS individuals. The study was carried out after ethical approval was obtained from the Whanganui-Manawatu human ethics committee.

### In Vitro Contracture Testing

*In vitro *contracture testing of muscle biopsies was performed according to the European Malignant Hyperthermia Group protocol [9].

### Primer design

Two allele-specific forward primers (AS primers; AS1 and AS2) were designed with the 3' base of each primer matching only the wild type or mutant H4833Y SNP, respectively. To ensure allele specificity, a mismatch two bases away from the 3'-end was added to each AS primer [25]. Both AS1 and AS2 had melting temperatures close to the annealing temperature (58°C). A common reverse primer with a higher melting temperature (65°C) was designed downstream of the polymorphic site and used together with either AS1 or AS2 in separate reactions. The AS-PCRs generated amplicons of 142 bp in size. The hypoxanthine-guanine-phosphoribosyltransferase (HPRT) [GenBank: M26434] gene was used as a reference, since it is expressed at lower levels as is *RYR*1, especially in LCLs. The primers for HPRT generated an amplicon of 85 bp in size. All the primer pairs are listed in Table 1 and span an intron to prevent genomic DNA (gDNA) amplification.

**Table 1:** Primer sequences

cDNA target	Orientation	Sequence (5'- > 3')
*RYR*1	Fw. (AS1)	CCATCCTGTCCTCTGTCATCC
	Fw. (AS2)	CCATCCTGTCCTCTGTCATCT
	Rev. (common)	GGTTCATCCTCATCCTCGCTCTTG

HPRT	Fw.	TCCAAAGATGGTCAAGGTCGC
	Rev.	TTCAAATCCAACAAAGTCTGGCT

Additional mismatches in the AS-PCR primer sequences are underlined.

### Allele-specific PCR conditions

All assays were carried out in 96-well format in 10 μL volumes and were performed using the following PCR cycling conditions. The PCR was initiated with a 15 min. hold at 95°C, followed by 45 cycles of 95°C for 10 s, 58°C for 10 s, and 72°C for 6 s. A single fluorescence measurement was conducted at the end of the 72°C extension segment. After heating the samples to 95°C for 5 s a melting curve program (65-97°C, with a ramp rate of 0.11°C/s and continuous fluorescence measurements), and finally a cooling step to 40°C were conducted. PCR crossing points (Ct values) were determined using the LightCycler 480 software. The reaction mixture for AS-PCR consisted of 0.3 μM of each primer, and 1 × ABsolute™ QPCR SYBR^® ^Green Capillary Mix (ABgene).

### Plasmid constructs

Engineered plasmids were used for assay validation and created by cloning 409 bp complementary DNA (cDNA) PCR fragments flanking the 4833 wild type and mutant RYR1 sequences into pGEM-T Easy vectors (Promega). The sequences of the cDNA forward and reverse primers were 5'-ACCTGGGCTGGTATATGGTG-3' and 5'-TGACGATGACGAAGAAG-3', respectively. DNA concentrations were estimated by A_260_. To assess primer specificity both wild type and mutant constructs were amplified with both AS1 and AS2. To test the possibility of using the designed AS-PCR protocol for relative quantification, four different ratios (1:1, 4:1, 3:1 & 1:2) of the engineered wild type and mutant plasmid constructs were mixed together in order to simulate heterozygotes.

### Extraction of total RNA

Total RNA was extracted from ~100 mg frozen skeletal muscle tissue or 5-10 × 10^6 ^cells using TRizol^® ^Reagent (Invitrogen) according to the manufacturer's instructions. Before reverse transcription, total RNA was treated with TURBO DNase (Ambion) according to the manufacturer's protocol.

### Linearity of the reverse transcription

The reverse transcription is a crucial step in quantitative analysis. To ensure that varying RNA amounts or RNA dilutions did not affect the reverse transcription reaction, the linearity of the reverse transcription reaction was checked for all samples. DNase treated total RNA from up to 1 μg for skeletal muscle and 2 μg for LCL was serial diluted four or five times in 1:5 or 1:3 steps, respectively. Due to the low RyR1 mRNA expression levels, dilutions included MS2 RNA (Roche; final concentration 10 ng/μL) in siliconized tubes to prevent binding to the sides of the tubes and thus improve reproducibility. Equal volumes of the serial diluted RNA were primed with Oligo(dT) and subjected to cDNA synthesis using the SuperScript™ III First-Strand Synthesis System for RT-PCR (Invitrogen) according to the manufacturer's protocol. All dilutions were amplified in triplicate with all three primer pairs (AS1, AS2 and HPRT) and the linear relationships of cDNA synthesis as a function of RNA content were determined by E = 10^-1/slope^. To verify the absence of contaminating cDNA or genomic DNA, reverse transcriptase was omitted from the cDNA synthesis reaction.

### PCR amplification efficiencies

PCR amplification efficiencies (E) of all three primers pairs (AS1, AS2 and HPRT) were determined by making a four or five times cDNA serial dilution in 1:5 or 1:3 steps, respectively. In contrast to artificial or cloned standards, these relative standards are more likely to have PCR efficiencies identical to the unknown samples as they are made from actual sample material. The dilutions were made including MS2 RNA (Roche; final concentration 10 ng/μL) in siliconized tubes to prevent binding to the sides of the tubes and thus improve reproducibility. The serial diluted cDNAs were amplified in triplicate. PCR amplification efficiencies were determined by constructing standard curves from the data of four individual experiments. PCR amplification efficiencies were determined by E = 10^-1/slope^.

### Establishment of lymphoblastoid cell lines

Peripheral mononuclear cells were isolated by Ficoll-Hypaque (Amersham Biosciences) density gradient centrifugation, from whole blood. The isolated mononuclear cells were then transformed with Epstein-Barr virus [26]. Cells were grown in OptiMem medium (Invitrogen) supplemented with 2% fetal calf serum (Invitrogen), and 100 U penicillin and streptomycin (Invitrogen), at 37°C, 5% CO_2_. Cells were cultured up to a total of 1-3 × 10^7 ^cells per 20 mL.

### mRNA stability assays

An mRNA stability assay was designed and carried out using LCLs to assess possible differences in mRNA stabilities between the two RyR1 alleles in heterozygous samples. Following the assessment of the linearity of the reverse transcription reactions and PCR amplification efficiency determinations (as described above), mRNA stability assays were conducted. After reaching the desired densities the cells were split into four and incubated with actinomycin D (Sigma-Aldrich) for 0, 2, 7 or 24 hours, at a final concentration of 5 μg/mL. Subsequently, total RNA was extracted and used for first-strand cDNA synthesis. cDNA was generated each time from the same amount of total RNA. The levels of all three targets (wild type *RYR*1: AS1, mutant *RYR*1: AS2 and HPRT) were then measured in real-time PCR assays.

## Results

### Assay validations using plasmid constructs

Both wild type and mutant plasmids were amplified with both AS1 and AS2 according to the conditions described above. The amplification curves had identical steep sigmoid curve shapes. Derivative melting curves generated only a single peak (Tm ~88°C), indicating that no non-specific products or primer-dimers were present. These results were confirmed by 1% agarose gel electrophoresis. Amplification of the wild type sequence by AS2 and amplification of the mutant sequence by the AS1 was delayed by at least 10 cycles, indicating appropriate specificities of the AS primers (data not shown).

To test the possibility of using the designed AS-PCR protocol for relative quantification, different ratios of the engineered wild type and mutant plasmid constructs were mixed together in order to simulate heterozygotes. Table 2 shows representative results as generated by a relative quantification experiment using plasmid constructs that contained either the wild type or mutant *RYR*1 cDNA sequences. Each sample was amplified in triplicate. The mean of the three Ct values was used to calculate the allele frequencies of that allele by using Equation 1 [27].(1)

**Table 2:** Relative allele frequencies using engineered plasmid constructs

Ratio (WT:MT)		Ct values				Percentage (%)	
		**Mean (n = 3)**	**Stdev**.	**ΔCt**	**^b^ΔΔCt**	**Theor**.	**Obs**.

4:1	AS1	18.26	0.09	-3.35	-2.10	80.00	81.09

	AS2	21.61	0.09	3.35	2.10	20.00	18.91

3:1	AS1	18.50	0.06	-2.72	-1.47	75.00	73.43

	AS2	21.21	0.08	2.72	1.47	25.00	26.57

1:2	AS1	19.55	0.07	-0.21	1.04	33.33	32.67

	AS2	19.76	0.04	0.21	-1.04	66.67	67.33

1:1	AS1	18.95	0.21	^a^-1.25	0.00	*-*	*-*

	AS2	20.20	0.08	^a^1.25	0.00	*-*	*-*

Stdev. = Standard deviation, Theor. = theoretical values, Obs. = experimentally observes values. ^a ^The ΔCt value for the 1:1 ratio should be equal to zero. Any deviation indicates differences in amplification efficiencies or DNA concentrations of the added plasmid constructs. Therefore the 1:1 ΔCt values were subtracted from the ΔCt values leading to ^b ^ΔΔCt. This value was used in Equation 1 to calculate the relative allele frequencies.

ΔCt = (Ct of allele_1 _specific PCR - Ct of allele_2 _specific PCR)

ΔΔCt = (Ct of allele_1 _specific PCR - Ct of allele_2 _specific PCR) - (ΔCt 1:1 ratio)

In order to correct for potential differences in amplification efficiencies or plasmid concentrations the 1:1 ΔCt value was subtracted from the 4:1, 3:1 and 2:1 ΔCt values. The resulting 2^ΔΔCt ^was used in Equation 1 to calculate the relative allele frequencies (see Table 2). The observed ratios matched the theoretical ratios with a maximum error of < 2%, indicating the applicability and sensitivity of the assay.

### Relative quantification of RyR1 transcripts in MHS samples

The corrections described above for differences in amplification efficiency and DNA concentrations are not possible using sample cDNA. Therefore, amplification efficiency determinations were conducted by making relative standards. Prior to amplification efficiency determinations, the linearity of the reverse transcription reaction was checked for all three targets. The amplification curves had identical steep sigmoid curve shapes and derivative melting curves generated only a single peak (Tm ~88°C), indicating that no non-specific products or primer-dimers were present. These results were confirmed by 1% agarose gel electrophoresis (data not shown). Table 3 summarizes the results of testing all four MHS skeletal muscle samples for linearity of reverse. The results revealed that the reverse transcription reactions of all RNA extracts were linear over the range used in this study. Thus, the cDNA library generated can be expected to be an appropriate representation of the mRNA populations in the samples.

**Table 3:** Summary of the linearity of the reverse transcription reactions

Muscle sample #	Amplification efficiencies and coefficient of correlation (E/R^2^)		
	**AS1**	**AS2**	**HPRT**

1	1.973/0.9957	1.913/0.9967	1.933/0.9990

2	2.074/0.9955	2.075/0.9956	2.032/0.9945

3	1.988/0.9986	1.873/0.9990	1.995/0.9923

4	1.976/0.9950	2.005/0.9908	2.107/0.9981

**Figure 1:**
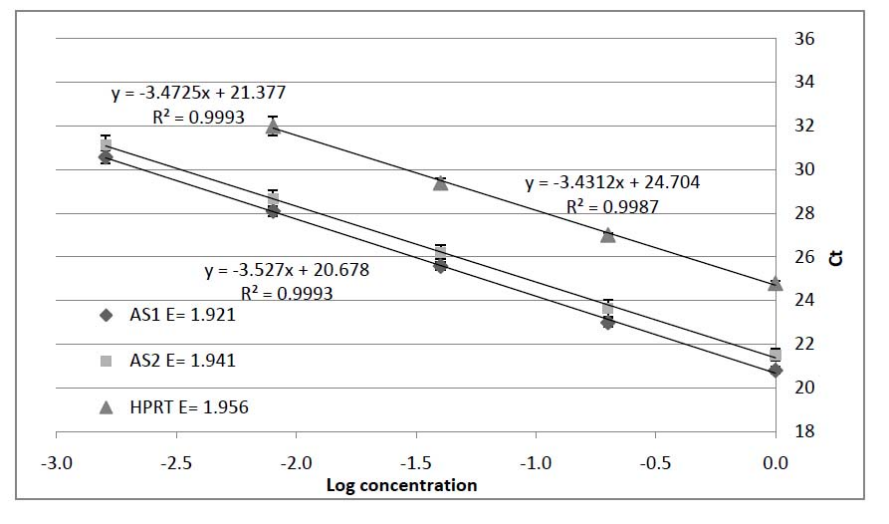
**Constructed standard curves for the determination of the PCR amplification efficiencies**. Serial diluted 1:5 cDNA dilutions from the #1 MHS muscle biopsy. Each standard curve represents the pooled results of four individual experiments. Amplifications in each individual experiment were conducted in triplicate. Housekeeping gene; HRPT (triangles), wild type *RYR*1; AS1 (diamonds) and mutant *RYR*1; AS2 (squares). The error bars show the standard deviation. The regression coefficient was greater than 0.99 in all cases.

PCR amplification efficiencies were calculated and used for the determination of the relative *RYR*1 mRNA allele frequencies. In order to correct for between-assay-variability, PCR amplification efficiencies were determined by constructing standard curves from the data of four individual experiments. Figure 1 shows the standard curves constructed for wild type *RYR*1 (AS1), mutant *RYR*1 (AS2) and HPRT. As the error bars indicate, assay variability was low. The "coefficient of variance" (CV%) of the Ct values, between triplicates (intra-assay variation) and between assays (inter-assay variation) was between 0.12% and 3% in each case. As could be expected, the higher CVs were generally associated with the more dilute samples. Only standard curves with slopes between -3.1 to -3.6 and regression coefficients > 0.99 were considered to be reliable. These slopes correspond to amplification efficiencies between 2.1 to 1.9, respectively. All three targets that were screened in the four samples fulfilled these requirements (see Table 4). The dilution series for each of the four samples were chosen according to their detected mRNA levels and the previously determined range of reverse transcription linearity. PCR amplification efficiencies of samples, in which fewer transcripts were detected, were determined by making 5 dilutions of 1:3 dilution steps instead 4 or 5 dilutions of 1:5 dilution steps.

**Table 4:** Mean PCR amplification efficiencies (n = 4)

Muscle sample #	Amplification efficiencies and coefficient of correlation (E/R^2^)		
	**AS1**	**AS2**	**HPRT**

1	1.921/0.9993	1.941/0.9993	1.956/0.9987

2	2.068/0.9989	2.104/0.9995	1.999/0.9995

3	2.030/0.9989	2.045/0.9996	2.028/0.9988

4	2.011/0.9986	2.079/0.9993	1.976/0.9895

Dilutions series used for the determination of the PCR amplification efficiencies for each of the four MHS muscle tissues: 1; 1:5 dilution steps, 2; 1:3 dilution steps, 3; 1:3 dilution steps, 4; 1:3 dilution steps.

### Determining the relative allele frequencies

After assessing the linearity of the reverse transcription reactions and determining the PCR amplification efficiencies, the samples were screened to determine the relative quantities of the two *RYR*1 alleles in each of the four samples. To confirm accuracy and reproducibility, the allele frequencies were determined by performing three independent experiments. Targets were amplified in triplicate within each experiment. Intra- and inter assay variabilities as obtained by screening sample #1 are shown in Table 5 and 6, respectively. As the results show, both intra- and inter assay variations are low.

**Table 5:** Intra-assay variability of real-time AS-PCR when screening #1 (n = 3)

Experimental run #	AS1			AS2			HPRT		
	**Mean Ct**	**Stdev**	**CV%**	**Mean Ct**	**Stdev**	**CV%**	**Mean Ct**	**Stdev**	**CV%**

1	23.66	0.13	0.54	24.08	0.10	0.40	27.21	0.12	0.42

2	23.65	0.06	0.06	24.13	0.02	0.09	27.16	0.05	0.19

3	23.53	0.27	1.14	24.08	0.11	0.11	27.09	0.12	0.43

Stdev. = Standard deviation, CV%= coefficient of variance.

**Table 6:** Inter-assay variability of real-time AS-PCR when screening #1 (n = 3)

Target	Mean Ct (n = 3)	**Stdev**.	CV%
AS1	23.61	0.07	0.31

AS2	24.10	0.03	0.14

HPRT	27.15	0.06	0.22

Stdev. = Standard deviation, CV%= coefficient of variance.

Several mathematical models are available for relative quantification during real-time PCR. There are some small differences between the individual models. Nevertheless, all relative quantification analyses are based on the assumption that the concentration of the template (e.g. cDNA) at a sample's crossing point is the same for every sample containing the same target cDNA. The model used in this study, is a rearranged version of the efficiency calibrated mathematical method (see Equation 2) [28]. In this model, calculations are based on E and the Ct values of an unknown "sample" versus a "calibrator". The "target" is the nucleic acid of interest, while the "reference" is a nucleic acid that is found at constant copy number in all samples and serves as an endogenous control. The "calibrator" is typically a sample with a stable ratio of target-to-reference and can be used to normalize all samples within a run, but in addition provides a constant calibration point between several runs.(2)

In this study the relative expression levels of all targets were measured simultaneously in one target tissue. When the two *RYR*1 alleles have identical mRNA expression levels it can be assumed that their HPRT:AS1 and HPRT:AS2 ratios will also be identical. Therefore, the two can be compared as long as the amplification efficiencies are corrected for. This eliminates the need for a calibrator, and if preferred also the reference. Here, the reference was still used as an endogenous control. As a result, the equation above was rearranged to Equation 3 and used for the calculations.(3)

Table 7 summarizes the results from the screens of the four muscle samples, using the H4833Y causative MH mutation as a marker. In all samples, expression of the wild type *RYR*1 allele (AS1) was found to be higher than the mutant *RYR*1 allele (AS2). In addition these values seem to differ between individuals. When the *RYR*1 mRNA expression ratios are compared with the data from the IVCT, no clear trend was detected.

**Table 7:** Relative RYR1 allele ratios in muscle tissues (n = 3)

Normalized ratios	Muscle sample			
**Mean ± stdev**.	**#1**	**#2**	**#3**	**#4**

Wild type RyR1 (CV%)	1.76 ± 0.08 (4.35%)	1.72 ± 0.27 (15.49%)	1.44 ± 0.14 (9.86%)	2.44 ± 0.40 (16.50%)

Mutant RyR1	1.00	1.00	1.00	1.00

**IVCT data**				

Maximum caffeine tension (g)	3.8	2.0	1.9	2.9

Maximum halothane tension (g)	3.2	6.6	2.6	7.0

Stdev. = Standard deviation, CV%= coefficient of variance.

### mRNA stability assays

Allele-specific differences in mRNA expression levels can have various causes (e.g. variations in mRNA transcription, maturation or stability). Myoblasts containing the H4833Y mutation were not available, thus immortalized LCLs that were derived from blood of MHS (H4833Y) individuals were used to assess the possibility that the detected allelic variations could be caused by allele-specific differences in *RYR*1 mRNA stabilities.

As for the muscle tissues, the linearity of the reverse transcription was determined for all three targets in LCLs. Due to the lower RyR1 expression in LCLs, amplification efficiencies were determined using five 1:3 dilution steps. Table 8 depicts the results of the reverse transcription linearity determinations in italics. As before, the results revealed that the reverse transcription reactions of all RNA extracts were linear over the range used in this study. Subsequently, PCR amplification efficiencies were determined from the data of four assays, and used for the determination of the relative *RYR*1 mRNA allele frequencies after actinomycin D incubations. The PCR amplification efficiencies of all three targets for both LCLs are underlined in Table 8.

**Table 8:** Linearity of the reverse transcription and PCR amplification efficiency determinations

LCL #	Amplification efficiencies and coefficient of correlation (E/R^2^)		
	**AS1**	**AS2**	**HPRT**

5	*1.882/0.9954*	*1.988/0.9953*	*2.059/0.9972*
	
	2.032/0.9975	2.039/0.9999	2.018/0.9997

6	*2.051/0.9965*	*2.007/0.9998*	*2.034/0.9994*
	
	2.010/0.9993	2.014/0.9978	2.019/0.9997

Determinations of the linearity of the reverse transcription reactions are depicted in italics. PCR amplification efficiency determinations are underlined.

mRNA stability assays were conducted by culturing LCLs in the absence or presence of actinomycin D for 0, 2, 7 or 24 hours, respectively. The levels of all three targets (wild type *RYR*1; AS1, mutant *RYR*1; AS2 and HPRT) were measured in by AS-PCR assays as described above. To confirm accuracy and reproducibility, all targets were measured in triplicate within each experiment (intra assay variability). Inter assay variability was addressed by conducting each individual experiment three times. To assess assay reproducibility the complete mRNA stability assays were repeated three times for both LCL #5 and #6. Reproducibility between the three assays was high, as Ct value CVs for both LCL #5 and #6, were < 3%. A clear increase in Ct values was detected after actinomycin D incubation after 24 hours, thus indicating a considerable decrease in initial mRNA levels, due to the inhibition of transcription see Additional file 1 and 2. For both LCLs (#5 and #6) the *RYR*1 mRNA levels decreased by > 86%. Note that the way the assay is conducted does not allow reliable detection of mRNA transcription after just two hours see Additional file 2. The mRNA levels of the housekeeping gene in each LCL, decreased by at least 57%. The relative amount of mRNA in the t = 24 h sample however, can be expected to be much higher compared to the relative amount of mRNA in the t = 0 h sample. This is because actinomycin D has a more pronounced effect on RNA polymerase I (ribosomal RNA synthesis) than it has on RNA polymerase II (mRNA synthesis) [29]. The observed decrease in mRNA transcripts was believed to be of an adequate range to allow for the detection of possible allele-specific differences between *RYR*1 mRNA stabilities.

After confirming transcriptional inhibition, the normalized ratios of the two RyR1 alleles were calculated, using Equation 3. Table 9 depicts the results for LCLs #5 and #6. As in muscle, in both samples the wild type *RYR*1 allele (AS1) was found to be more abundant than the mutant *RYR*1 allele (AS2). For both LCLs the ratios between the wild type and mutant *RYR*1 alleles did not change significantly after different incubation times with actinomycin D. Note that the ratios between the wild type and mutant *RYR*1 mRNA expression levels differ between LCLs.

**Table 9:** Relative *RYR*1 allele ratios of LCLs after incubation with actinomycin D

Normalized ratios LCL #5	**Actinomycin D incubation time (h) mean ± stdev**.			
	**0**	**2**	**7**	**24**

Wild type RYR1 (CV%)	1.22 ± 0.25 (20.70%)	1.44 ± 0.24 (16.83%)	1.43 ± 0.22 (15.17%)	1.12 ± 0.07 (6.24%)

Mutant RYR1	1.00	1.00	1.00	1.00

**Normalized ratios LCL #6**				

Wild type RYR1 (CV%)	1.86 ± 0.28 (15.21%)	1.82 ± 0.15 (8.14%)	1.86 ± 0.43 (23.38%)	1.67 ± 0.17 (10.05%)

Mutant RYR1	1.00	1.00	1.00	1.00

Stdev. = Standard deviation, CV%= coefficient of variance.

## Discussion

Allele-specific expression has been shown to be common in the human genome even among non-imprinted autosomal genes and can play a major role in determining phenotypic diversity [23]. Fluorescent dideoxy terminators [30], matrix-assisted laser desorption/ionization time-of-flight mass spectroscopy (MALDI-TOF MS) [31], micro-arrays (Affymetrix HuSNP oligo arrays) and real-time PCR using TaqMan probes [23] are several methods that have been used for allele-specific expression analysis.

In this report, an assay was developed that combined kinetic (real-time quantitative) PCR with allele-specific amplification to determine the relative mRNA quantities of the two *RYR*1 alleles in heterozygous MHS samples. The causative H4833Y MH mutation present in the coding region of the *RYR*1 gene was used as a marker to distinguish between the two alleles. Unlike many other real-time PCR methods, AS-PCR does not need expensive fluorescently-labelled oligonucleotides. Instead SYBR Green was used as an inexpensive generic fluorescent DNA-binding dye.

Assay validations conducted using engineered plasmid constructs revealed that both AS primers (AS1 and AS2) were highly specific for either the wild type or mutant RyR1 allele, respectively. A delay of at least 10 cycles was observed upon non-specific binding. The possibility of using the designed AS-PCR protocol for relative quantification was tested by mixing the engineered wild type and mutant plasmid constructs together in four different ratios (1:1, 4:1, 3:1 and 1:2). The results indicated the applicability and sensitivity of the AS-PCR assay as the observed ratios matched the theoretical ratios with a maximum error of < 2% (see Table 2).

Screening of the four MHS muscle samples revealed allele-specific differences in *RYR*1 mRNA expression levels (see Table 7). In all four samples the wild type (H4833) allele was found to be expressed at higher levels than the mutant *RYR*1 allele (Y4833). Note however, that it is not necessarily the mutant RyR1 allele that is down-regulated. It is possible that the cell compensates for the defective RyR1 allele by increasing transcript levels of the wild type RyR1 allele. In three (#1, #2 & # 4) out of four samples the H4833Y MH mutation could be traced back and was found to be inherited from the mother. The calculated averages of the relative RyR1 allelic ratios of three independent assays led to CV% values of < 17%, in all cases. These slightly higher CV% values compared to Ct value CV% are the result of the fact that the Ct values are exponentially related to initial mRNA concentrations. When the detected allelic ratios were compared with data from the IVCT (see Table 7) no clear trend was detected. This is not unexpected as the SR calcium release is a finely regulated process that involves not only RyR1s. Several other genetic and environmental factors can contribute to the observed muscle contractions in the IVCT (metabolic processes, enzyme activation, muscle composition, differences in gene expression). Furthermore, the RyR1 is comprised of four identical subunits. In each tetramer, any of the individual RyR1 subunits can be defective and thus contribute to the observed variations in MH phenotypes. In addition only every other RyR1 tetramer is associated with a DHPR tetrad [32].

As H4833Y myoblasts were not available, LCLs were used for mRNA stability assays using the transcription inhibitor actinomycin D. LCLs are progressively being used to assess the role of RyR1 mutations in calcium release [11,13]. Possible allelic variations in RyR1 expression levels in LCLs can greatly affect the functional characterization of potentially causative MH mutations. As for skeletal muscle tissue, in each of the two LCLs (#5 and #6) the wild type RyR1 allele (AS1) was found to be more abundant than the mutant RyR1 allele (AS2; see Table 9). In addition, the ratios between the wild type and mutant *RYR*1 mRNA expression levels differ between LCLs. This suggests that individual specific genetic and or environmental factors also contribute to the relative expression levels of wild type and mutant RyR1. In all cases, CV% values of < 24% were obtained based on the calculated averages of the relative *RYR*1 allelic ratios of three independent experiments. These slightly higher CV% values can be expected and are the result of the lower *RYR*1 mRNA expression levels in LCLs. For both LCL samples, the ratios between the wild type and mutant *RYR*1 transcripts did not change after different incubation times with actinomycin D. This suggests that there are no allele-specific differences in RyR1 mRNA stability, at least in LCLs. Nevertheless, allelic variation has been reported to be tissue specific [22,33]. Thus, the effect of allele-specific differences in RyR1 mRNA stabilities in H4833Y MHS skeletal muscle tissues cannot be ruled out. Alternatively the observed allelic variation could be the result of variations in mRNA transcription rates or mRNA maturation.

The cause of the observed allelic variations is as yet unknown. Nevertheless, the mechanism seems to be distinct from the one previously reported by Zhou, et al. [34]. In that study, tissue-specific mono-allelic *RYR*1 expression was observed in a group of CCD patients with recessive core myopathies. The transcribed allele which was found to be paternally inherited carried a recessive mutation. Additional analyses indicated that *RYR*1 allele silencing was also tissue-specific and polymorphic during early development and likely to be developmentally regulated. It was hypothesized that genomic imprinting due to long-term methylation effects could be responsible and possibly also explain heterogeneity between MH phenotypes [33,34]. Recently Robinson *et al*. investigated the possibility of epigenetic *RYR*1 silencing and variable penetrance of MH susceptibility. Out of 2113 transmissions, it was found that affected (MHS or MH equivocal; MHE) fathers had significantly fewer affected daughters than affected sons or unaffected (MH negative; MHN) daughters. Nevertheless, no discrepancies were observed between genotypes at the gDNA and cDNA level suggesting the absence of mono-allelic *RYR*1 expression in MH [35]. Note however that unlike the mono-allelic expression associated with e.g. genomic imprinting, the detection of possible minor allelic variations in gene expression requires more quantitative detection methods.

## Conclusion

The data presented here revealed for the first time allele-specific differences in RyR1 mRNA expression levels in heterozygous MHS samples. The detected allelic variation in RyR1 mRNA expression levels could at least in part contribute to the observed variable penetrance and variations in MH phenotypes [19-21]. Whether or not these allele specific-differences translate through to the protein levels remains to be elucidated.

## Competing interests

The authors declare that they have no competing interests.

## Authors' contributions

HG drafted the manuscript, conducted, analysed the data and made substantial contributions to concept and design. KS devised the study and participated in drafting the manuscript. All authors read and approved the final manuscript.

## Supplementary Material

Additional file 1 **Time course of *RYR1 *mRNA expression levels after actinomycin D incubation in LCL #5.** Initial mRNA expression levels were measured in real-time PCR after LCLs were incubated for different times with the transcriptional inhibitor actinomycin D. Each curve represents the pooled results of three independent mRNA stability assays. Housekeeping gene; HRPT (triangles), wild type *RYR*1; AS1 (diamonds) and mutant *RYR*1; AS2 (squares). The error bars show the standard deviation. Click here for file

Additional file 2 **Time course of *RYR1 *mRNA expression levels after actinomycin D incubation in #6.** Initial mRNA expression levels were measured in real-time PCR after LCLs were incubated for different times with the transcriptional inhibitor actinomycin D. Each curve represents the pooled results of three independent mRNA stability assays. Housekeeping gene; HRPT (triangles), wild type *RYR *1; AS1 (diamonds) and mutant *RYR *1; AS2 (squares). The error bars show the standard deviation. Click here for file
