# Supplementary figures and images for: Allele-specific differences in ryanodine receptor 1 mRNA expression levels may contribute to phenotypic variability in malignant hyperthermia
Source: Orphanet J Rare Dis. 2010 May 19;5:10. doi: 10.1186/1750-1172-5-10 (PMC2895584; doi:10.1186/1750-1172-5-10)

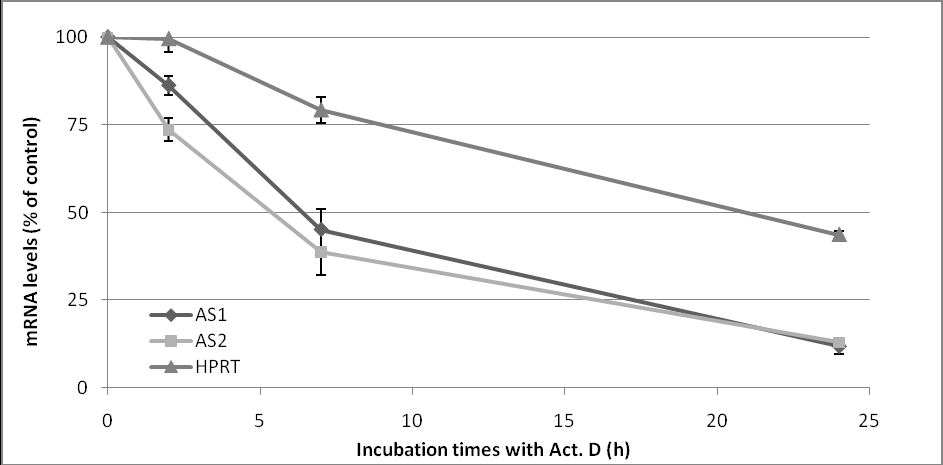

Supplement: Additional file 1 — Time course of RYR1 mRNA expression levels after actinomycin D incubation in LCL #5. Initial mRNA expression levels were measured in real-time PCR after LCLs were incubated for different times with the transcriptional inhibitor actinomycin D. Each curve represents the pooled results of three independent mRNA stability assays. Housekeeping gene; HRPT (triangles), wild type RYR1; AS1 (diamonds) and mutant RYR1; AS2 (squares). The error bars show the standard deviation. [file 1750-1172-5-10-S1.TIFF]

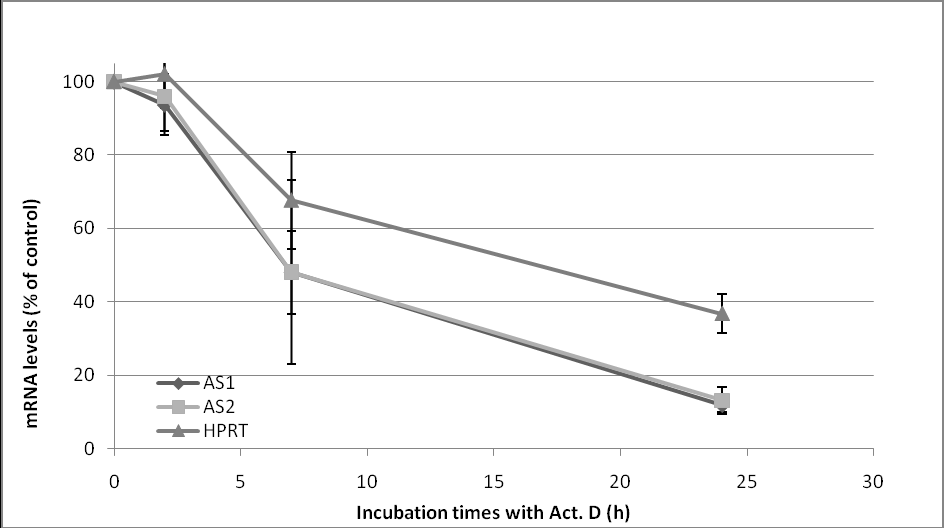

Supplement: Additional file 2 — Time course of RYR1 mRNA expression levels after actinomycin D incubation in #6. Initial mRNA expression levels were measured in real-time PCR after LCLs were incubated for different times with the transcriptional inhibitor actinomycin D. Each curve represents the pooled results of three independent mRNA stability assays. Housekeeping gene; HRPT (triangles), wild type RYR 1; AS1 (diamonds) and mutant RYR 1; AS2 (squares). The error bars show the standard deviation. [file 1750-1172-5-10-S2.TIFF]
